# Safety and durability of AGT103-T autologous T cell therapy for HIV infection in a Phase 1 trial

**DOI:** 10.3389/fmed.2022.1044713

**Published:** 2022-11-14

**Authors:** Nidal Muvarak, Haishan Li, Tyler Lahusen, Jeffrey A. Galvin, Princy N. Kumar, C. David Pauza, José Bordon

**Affiliations:** ^1^American Gene Technologies International, Inc., Rockville, MD, United States; ^2^Georgetown University School of Medicine, Washington, DC, United States; ^3^Washington Health Institute, Washington, DC, United States

**Keywords:** HIV, cell therapy, gene therapy, immune reconstitution, functional cure

## Abstract

The cell and gene therapy product AGT103-T was designed to restore the Gag-specific CD4+ T cell response in persons with chronic HIV disease who are receiving antiretroviral therapy. This autologous, genetically engineered cell product is under investigation in a Phase 1 clinical trial (NCT03215004). Trial participants were conditioned with cyclophosphamide approximately 1 week before receiving a one-time low (< 10^9^ genetically modified CD4+ T cells) or high (≥10^9^ genetically modified CD4+ T cells) dose of AGT103-T, delivering between 2 and 21 million genetically modified cells per kilogram (kg) body weight. There were no serious adverse events (SAEs) and all adverse events (AEs) were mild. Genetically modified AGT103-T cells were detected in most of the participant blood samples collected 6 months after infusion, which was the last scheduled monitoring visit. Peripheral blood mononuclear cells (PBMC) collected after cell product infusion were tested to determine the abundance of Gag-specific T cells as a measure of objective responses to therapy. Gag-specific CD4+ T cells were detected in all treated individuals and were substantially increased by 9 to 300-fold compared to baseline, by 14 days after cell product infusion. Gag-specific CD8+ T cells were increased by 1.7 to 10-fold relative to baseline, by 28 days after cell product infusion. Levels of Gag-specific CD4+ T cells remained high (~2 to 70-fold higher relative to baseline) throughout 3–6 months after infusion. AGT103-T at low or high doses was safe and effective for improving host T cell immunity to HIV. Further studies, including antiretroviral treatment interruption, are warranted to evaluate the product's efficacy in HIV disease.

**Clinical trial registration:**

www.clinicaltrials.gov, identifier: NCT03215004.

## Introduction

Chronic HIV disease is characterized by dysregulation of host immunity through a variety of mechanisms including cell depletion. Among the most critical pathogenetic events is severe depletion of Gag-specific CD4+ T cells and levels of these cells in blood were negatively correlated with viremia (1–3). Rare individuals (natural or elite controllers) who suppress HIV replication without antiretroviral therapy are characterized by persistent, high levels of Gag-specific CD4+ T cells (4, 5) including HIV-specific mucosal CD4+ T cells (6). Natural virus control was correlated with Gag-specific CD4+ T cell proliferative responses independently of the CD8+ CTL response to HIV (7). Consequently, the loss of Gag-specific CD4+ T cell responses is a marker of HIV disease, highly correlated with both viremia and natural virus control, and a strong target for therapeutic intervention.

Correcting the Gag-specific CD4+ T cell deficit might be accomplished through cell and gene therapy, including the use of genetically engineered T cell products such as AGT103-T, which are highly enriched in Gag-specific CD4+ T cells. The challenge to making cell products capable of restoring antiviral CD4+ T cells is the duplicitous nature of these cells. On one hand, Gag-specific CD4+ T cells are required to orchestrate potent antiviral immune responses needed to contain virus replication. In an opposing role, they are highly susceptible to virus and support efficient HIV replication (8, 9). In the AGT103-T cell product, we sought to exploit the potency of Gag peptide stimulation to enrich the cell product and increase cellular immunity, while engineering cells with an antiviral lentivirus vector to inhibit virus infection and replication.

Design and preclinical evaluation of AGT103-T were reported previously (10) and are summarized here. The drug product has two components. One is the third generation, self-inactivating lentivirus vector AGT103. This vector expresses a cluster of three engineered microRNA (miRNA) under control of the constitutive EF1α promoter. The miRNA cluster was designed to decrease RNA levels through recognition of target sequences in mRNA encoding the host protein CCR5, and in genomic or sub-genomic RNA sequences from the HIV Tat and Vif genes. CD4+ T cell lines and primary cells transduced with AGT103 exhibited potent resistance against HIV infection. Because the vector incorporates miRNA targeting viral sequences in addition to targeting CCR5, transduced cells are protected from both R5- and X4-tropic strains of HIV (10). The second component in the drug product is CD4+ T cells derived from peripheral blood, in which the Gag-specific CD4+ T cell subset has been enriched by stimulating PBMC with synthetic peptides representing the HIV-1 Gag polyprotein. Unique features of the HIV Gag protein, including the presence of immunodominant epitopes such as Gag 293 (11), the abundance of public clonotypes in the CD4+ T cell repertoire of Gag-specific cells (12), and extensive cross-restriction of promiscuous epitopes by multiple, unrelated donors (13) allowed for consistent responses to Gag peptides irrespective of genetic background. After peptide stimulation, the activated cells were transduced with AGT103 lentivirus vector and grown in medium containing the protease inhibitor Saquinavir to prevent residual virus replication.

AGT103-T cell therapy will increase the population of Gag-specific CD4+ T cells, which are critically low in HIV+ individuals (14), highly susceptible to HIV infection (8, 9) and exhibit shorter life spans reflecting their increased viral burden (15). Numerous studies defined the critical relationship between Gag-specific CD4+ T cell levels and clinical status (3, 16, 17). In contrast, Gag-specific CD8+ T cell levels were only weakly correlated with viremia or clinical status and CD4+ T cell responses to the envelope glycoprotein were negatively associated with clinical status (2). Reconstituting Gag-specific CD4+ T cells and inhibiting their destruction through genetic modification should enhance viral immunity, control HIV viral burden, and reduce or eliminate the need for ART (18). Thus, this unique immunotherapeutic product has the potential to reconstitute a key component of HIV immunity, namely Gag-specific CD4+ T cells, using cells capable of performing all the functions expected of this subset and engineered to resist HIV-mediated depletion should they again encounter the virus. Through this strategy we hope to repair viral immunity and restore the capacity for virus suppression with modified antiretroviral therapy (ART) or in the absence of drug therapy.

After review by the US Food and Drug Administration (FDA) and approval from institutional review boards (IRBs), we initiated a Phase 1 trial to evaluate safety and feasibility of AGT103-T cell infusion in HIV+ individuals with well-controlled HIV on ART. The primary endpoints concerned safety of the cell product. The secondary endpoints included measuring objective responses to treatment, persistence of genetically modified CD4+ T cells, CD4+ T cell responses to Gag peptide stimulation, and CD8+ T cell responses to Gag peptide stimulation.

## Methods

### Participants

To date, a total of thirteen (13) participants were enrolled in the Phase 1 clinical trial (NCT03215004). The median age of participants was 41 years (range, 26–59). Median absolute CD4+ T cell count per microliter at screening was 577 cells per microliter (range, 437–1,465 cell per microliter). Duration of HIV infection (from time of diagnosis) ranged from 3.8 to 28.4 years (median, 14.2 years) and number of years on ART regimen ranged from 3 to 24 years, with a median of 6 years (Table 1). The participants included 11 adult males and 1 adult female. We are working to improve recruitment of female trial participants.

**Table 1:** Demographics of participants enrolled in Phase 1 study.

**PID^*a*^**	**Gender**	**Age**	**Race**	**Duration of HIV infection (years)**	**# Of years on ART regimen**	**CD4+ T cell (cells/μL)**
01-002	M	35	Black	15.8	4	942
01-005	M	47	White	10	10	693
01-007	M	26	White	3.8	3	577
01-006	M	36	Black	5.9	5	640
02-001	M	47	White	15.1	5	467
02-002	M	33	White	4.1	6	1,465
02-003	M	33	White	14.2	14	541
02-005	M	46	White	20.4	20	503
01-008	M	41	Black	5.7	6	760
02-008	M	33	White	4.2	4	1,577
01-010	F	46	Black	22.3	22	437
02-009	M	54	Black	28.4	24	496
02-010	M	59	White	17.2	14	484

^*a*^PID, participant ID.

### Cell manufacturing

The 12-Day cell manufacturing process was reported previously (10). All cell products were manufactured at Minaris Regenerative Medicine, LLC (Allendale, NJ). PBMC from leukapheresis packs (approximately 100–150 mL) were stimulated with a mixture of overlapping, synthetic peptides covering the HIV-1 Gag polyprotein (PepMix HIV-1 GAG; JPT) and cultured in medium containing the protease inhibitor Saquinavir that was included in all subsequent cell culture steps. Stimulated cells were enriched by negative selection for CD4+ T cells and transduced with lentivirus vector AGT103 in a CliniMACS Prodigy machine (Miltenyi Biotec). The stimulated and transduced cells were transferred to a G-Rex 500M-CS container (Wilson Wolf) for 8 days of static culture to expand the Gag-specific CD4+ T cell subset. The final drug product was collected, concentrated, washed to remove Saquinavir and growth medium, then resuspended in cryopreservation medium before storing at ultralow temperature to preserve cell viability. Manufacturing materials, equipment and procedures conformed to the rules for Good Manufacturing Processes (GMP).

### Clinical product release

Upon completing the manufacturing of each AGT103-T product, quality control (QC) samples were removed for release testing and the remaining drug product was cryopreserved until testing was completed and the product was deemed ready for infusion. QC lot release criteria (Table 2) included measuring the total number of transduced cells; this criterion defined the clinical dose. Low dose drug products contained ≥1 × 10^8^ and < 1 × 10^9^ AGT103-transduced, CD4+ T cells. High dose drug products contained ≥1 × 10^9^ and < 5 × 10^9^ AGT103-transduced CD4+ T cells. The HIV protease inhibitor Saquinavir was included in all steps of manufacturing to prevent spread of HIV (10). Although HIV was inhibited by Saquinavir, it was necessary to develop and qualify an HIV infectivity assay as part of product release testing. The assay used culture media from the final step in cell product manufacturing, to inoculate indicator cells that were cultured *in vitro*. All samples were negative for infectious HIV. Testing for replication competent lentiviruses (RCL), a potential contaminant in transduced cell mixtures, was completed in standard cell-based RCL assays (19) plus two custom assays for detecting transgene mobilization to rule out potential spread of RCL that may arise by recombination between the vector and HIV sequences present in cells of infected individuals. These rigorous QC lot release assays ensured that AGT103-T drug products could be released for participant infusion.

**Table 2:** Drug product release testing and specifications.

**Product attribute**	**Test**	**Specification**
Identity	Vector copy number (VCN)	0.01–5.0 copies per cell
Identity	Total number of transduced cells	0.1–5.0 x 10^9^ cells
Identity	VCN per transduced cell	< 5 VCN/transduced cell
Identity	Frequency of Gag-specific CD4 T cells	Report results
Identity	Frequency of CD3 and CD4 cells	CD3: ≥80%; CD4: ≥50%
Purity	Cell viability	≥70%
Microbial safety	Endotoxin	< 1 EU/mL
Microbial safety	Sterility	Negative
Microbial safety	Mycoplasma	Negative
Microbial safety	Replication competent lentivirus (RCL)	Not detected
Microbial safety	HIV infectivity	Not detected (CPE)^*a*^; ≤ LLOQ^*b*^ (Vif RT-qPCR)
Microbial safety	Transgene mobilization	Not detected

^*a*^CPE, cytopathic effect.

^*b*^LLOQ, lower limit of quantitation.

Of the thirteen enrolled participants who underwent leukapheresis, eleven products were produced and two products failed due to deviations in manufacturing. One product expired and three products were damaged during storage and not suitable for infusion. Seven products were infused during the Phase 1 clinical trial.

### Clinical trial design

**Figure 1:**
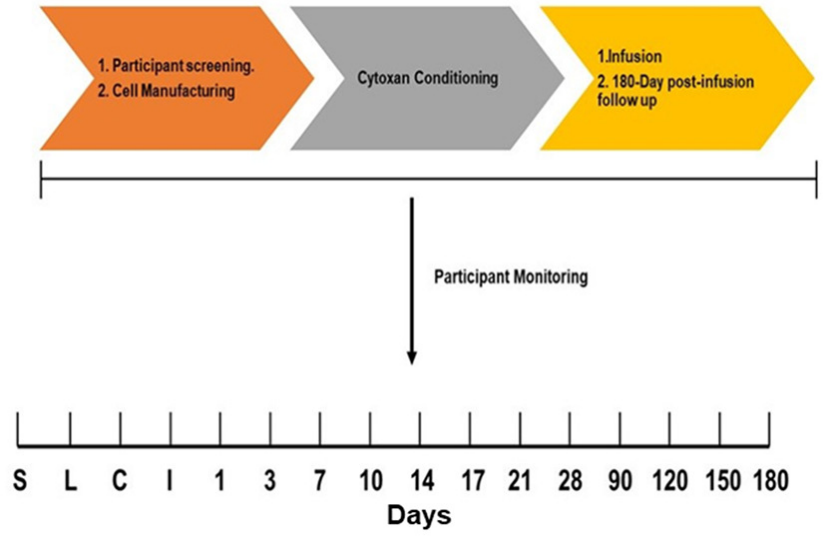
Clinical trial overview and participant monitoring schedule. **Upper panel**: Phase 1 study starts with informed consent and participant screening. Once participant eligibility is confirmed, the participant is scheduled for leukapheresis for cell manufacturing. 7 days prior to infusion, participants are conditioned with Cytoxan (cyclophosphamide) to enhance engraftment, then infused with AGT103-T. **Lower panel**: After screening (S), participants undergo leukapheresis (L) for AGT103-T manufacturing. When ready, participants are conditioned (C) with Cytoxan and infused (I) 7 days later. The participants were monitored during 1–180 days after infusion.

An overview of the clinical trial design is presented in Figure 1. The trial was initiated after IRB approval and participant informed consent. Participants were screened and scheduled to undergo leukapheresis for cell manufacturing as described above. When QC release testing was completed and drug products were ready for infusion, participants were scheduled for non-myeloablative conditioning with a single dose of cyclophosphamide (1 g/m^2^) approximately 7 days prior to infusion. Cyclophosphamide (Cytoxan) conditioning was used to enhance cell product engraftment as reported previously (20, 21). After the conditioning period, participants were infused with their autologous AGT103-T cell product, starting with the low dose products. Infusions of AGT103-T for the first three participants were separated by 45-day intervals to allow safety observations. After approval by the Data and Safety Monitoring Board (DSMB), the 45-day period was removed, and low dose products were infused after completion of QC release testing. In addition, high dose infusions commenced after the first three low-dose products, also separated by 45 days for the first high dose products, until approval was granted by the DSMB to infuse high dose products as they became available. After infusion, participants were monitored for 4 h, and scheduled for follow up visits for up to 180 days (Figure 1, lower panel).

### Assessments of safety

Adverse events were graded following the National Cancer Institute's Common Terminology Criteria for Adverse Events, version 5 (CTCAE, v5).

### Detecting AGT103-modified T cells

Assays for detecting the frequency of transgene-containing AGT103-T cells were conducted at the University of Pennsylvania Translational and Correlative Studies Laboratory as described previously (22, 23). Briefly, whole blood was collected during visits in K_2_EDTA tubes (BD Biosciences), which were then processed by Ficoll (GE) separation to isolate PBMC. Total DNA was extracted from PBMC pellets (approximately 1 million cells) for determining transgene copy number by quantitative polymerase chain reaction (qPCR) targeting sequences in the woodchuck hepatitis virus posttranscriptional response element (WPRE), which is found in the majority of lentiviral vectors including AGT103. Copy number was expressed as the average markings (transgene) per cell for AGT103-T cell products, and as average markings per microgram (μg) DNA, which was converted to average number of copies per million PBMC as described (23).

### Detecting HIV-specific CD4+ and CD8+ T cells

Whole blood was collected in Lithium heparin tubes and PBMC were isolated using Sepmate tubes (StemCell Technologies) per manufacturer's instructions. PBMC were cryopreserved in RPMI (ThermoFisher Scientific) containing 5% human AB serum (hABS, MilliporeSigma) and 7% DMSO (MilliporeSigma) until ready for analysis. Cyropreserved PBMC vials were viably thawed, and cells were rested for 18–22 h in RPMI + 5% hABS. The following day, cells were counted and resuspended in fresh RPMI + 5% hABS and stimulated in the presence of GolgiPlug (1:1,000, BD Biosciences) with 1 μg/mL PepMix HIV-1 GAG Ultra (JPT Peptide Technologies) for 4 h at 37°C and 5% CO2. Medium containing DMSO was used as a negative control for stimulation, and CEF/CPI Class I/II- restricted peptides (Cellular Technology Limited, CTL) were used as positive controls for responses to common antigens. After stimulation, cells were collected and washed in Dulbecco's phosphate buffered saline (DPBS, ThermoFisher Scientific) and stained with Fixable Viability Stain 450 (BD Biosciences) to exclude dead cells. Cells were washed in FACS staining buffer, blocked in TruStain Fc blocking solution (Biolegend) then stained with anti CD3-PerCP, CD4-FITC, and CD8-PE (Biolegend) antibodies, followed by fixation using 4% paraformaldehyde (EMS). Fixed cells were permeabilized in 1X prem/wash buffer (BD Biosciences) followed by intracellular staining with IFNγ-APC antibody (Biolegend). Cells were washed with perm/wash buffer and stored in FACS staining buffer until ready for analysis using a BD FACSLyric flow cytometer. Data analysis was performed using Flowjo software (BD Biosciences). Frequencies of HIV Gag-specific CD4+ and CD8+ T cells were expressed as the number of Gag-specific cells per million CD3+ T cells.

### Statistical evaluation

Microsoft Excel was used to calculate median values and percentages. Frequencies of Gag-specific T cells (# per million CD3+ cells) were calculated by multiplying percentages produced in Flowjo by 10,000. Fold-change was determined by normalizing number of post-infusion Gag-specific T cells to their numbers at baseline. All calculations and formulas were produced in Microsoft Excel.

## Results

### Primary endpoints

The primary objective of the Phase 1 study is to evaluate the safety of AGT103-T infusion in HIV+ participants with well-controlled viremia who are on ART. A total of 13 individuals (Table 1) were enrolled. Two cell manufacturing runs were aborted due to manufacturing errors. Three successfully manufactured products could not be infused due to failures during storage (compromised cryobags). One cell product expired before the participant could be infused. Infusions completed to-date are listed in Table 3. Low-dose and high-dose AGT103-T were infused in five and two participants, respectively. Doses, defined by number of AGT103-transduced cells, ranged from 2.00 × 10^6^ to 21.33 × 10^6^ cells per kg (median 6.68 × 10^6^ cells per kg). No serious adverse events (AEs) were reported for participants infused with AGT103-T. All reported AEs (Table 4) were mild; 14 out of 16 AEs were classified grade 1, and 2 out 16 AEs were classified as grade 2. The most common AE, related to cyclophosphamide conditioning, was nausea. Other low-grade AEs related to AGT103-T infusions included headache and body aches, both resolved within 1–2 days. Two grade-1 AEs (constipation and bruising at site of administration) lasted approximately 1 week before they resolved.

**Table 3:** AGT103-T cell product infusions.

**PID^*a*^**	**Dose category**	**Dose^*b*^**
01-002	Low	3.36 x 10^6^
01-005	Low	4.93 x 10^6^
01-007	Low	2.00 x 10^6^
01-006	Low	12.48 x 10^6^
02-001	Low	6.68 x 10^6^
01-008	High	21.33 x 10^6^
02-009	High	15.99 x 10^6^

^*a*^Participant Identification. ^*b*^Defined as number of genetically modified cells per Kg body weight.

**Table 4:** Adverse events.

**AE**	Procedure*	**AE grade**	**Incidence**	**AGT103-T dose**
Itchy throat	I	1	1/7 (14%)	Low
Body ache	I	1	2/7 (29%)	Low
Chills	C	2	1/7 (14%)	Low
Headache	I	1	3/7 (43%)	Low (*n* = 6) High (*n* = 1)
Malaise	I	1	1/7 (14%)	Low
Constipation	C	1	1/7 (14%)	Low
Nausea	C	1 and 2	3/7 (43%; Gr1); 1/7 (14%; Gr2)	Low (*n* = 3); High (*n* = 1)
Decreased appetite	C	1	1/7 (14%)	Low
Administration site bruise	I	1	1/7 (14%)	Low
Dysgeusia	I	1	1/7 (14%)	Low
Pyrexia	I	1	1/7 (14%)	Low

^*^C, cyclophosphamide conditioning; I, AGT103-T cell infusion.

**Figure 2:**
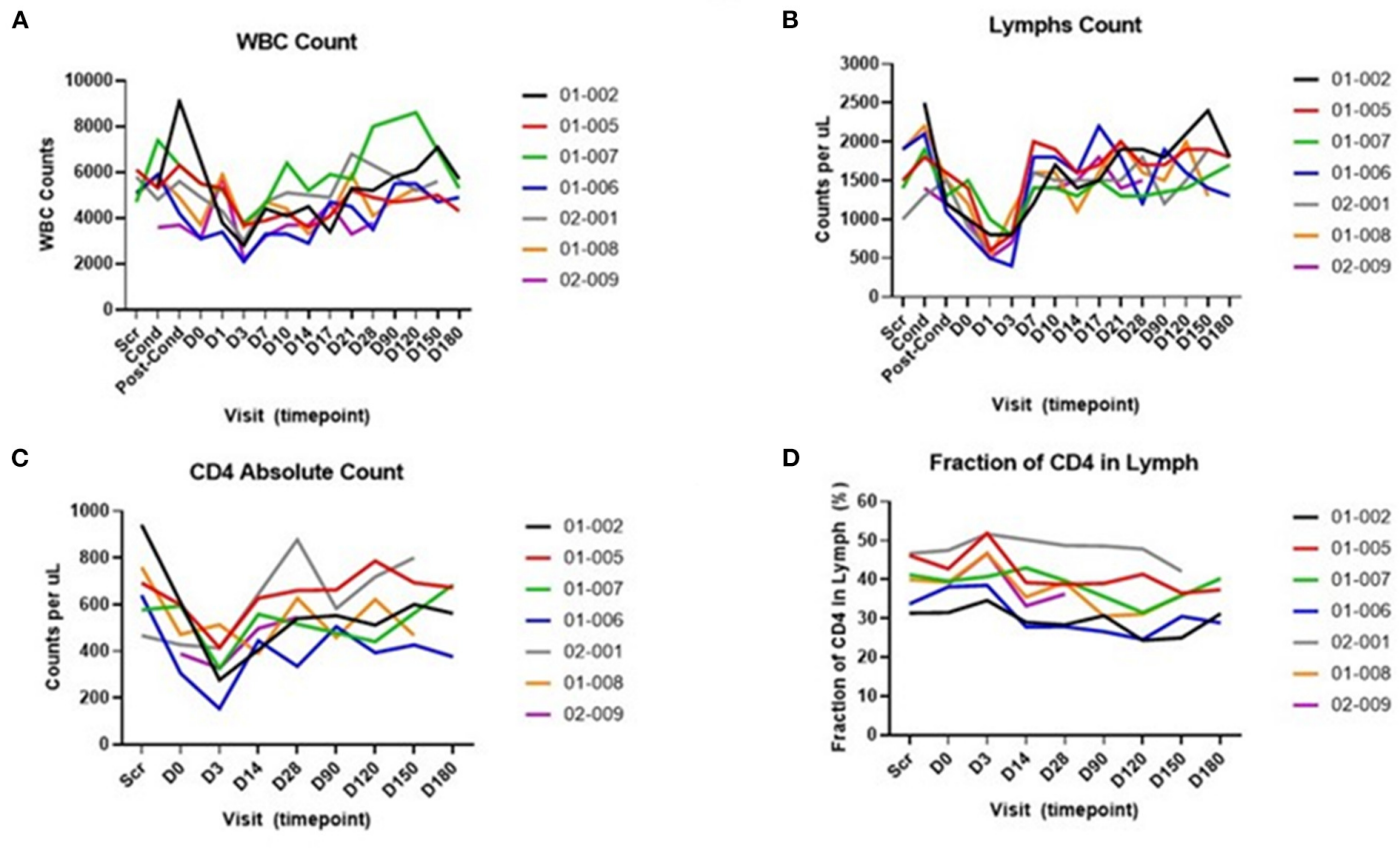
Hematology studies during participant monitoring. **(A)** White blood cell (WBC) counts expressed as counts per microliter (μL). **(B)** Lymphocyte (lymphs) counts expressed as counts per microliter (μL). **(C)** Absolute CD4 T cells, expressed as counts per microliter (μL). **(D)** Fraction of CD4 T cells in lymphocytes, expressed as a percentage (%). Scr, screening; cond, cyclophosphamide conditioning; post-cond, post-conditioning, D0 indicates the day of infusion; D1, D3, etc., indicate days after infusion. Individual trial participants are indicated by a 5-digit identifier in the legend.

No AEs were reported from laboratory studies (hematology and chemistry). Participants' white blood cell (WBC) and lymphocyte counts initially decreased because of conditioning but returned to pre-treatment levels (Figures 2A,B). Absolute CD4 counts followed a similar trend (Figure 2C), with exception of one participant (01-002) who had a sustained decrease in absolute CD4+ T cells compared to their screening visit. However, their fraction of CD4+ T cells in total lymphocytes returned to screening levels (Figure 2D). Taken together, conditioning and infusion of AGT103-T were completed successfully and safely without any serious AEs or risks for participants.

### Secondary endpoints

In addition to safety and feasibility of AGT103-T infusion, we characterized each cell product in terms of the total number of transduced cells, average vector copy number as a measure for lentivirus vector transduction, and the final proportion of Gag-specific CD4+ T cells (Table 5).

**Table 5:** Characteristics of individual AGT103-T cell products in the Phase I clinical trial.

**PID^1^**	**Dose**	**Total**	**VCN^2^**	**% Gag-**
	**category**	**number of**		**specific**
		**transduced**		**CD4 T cells**
		**cells**		**in Lymph**
01-002	Low	192 × 10^6^	0.07	13.0
01-005	Low	460 × 10^6^	0.10	10.3
01-007	Low	190 × 10^6^	0.09	1.2
01-006	Low	780 × 10^6^	0.09	29.3
02-001	Low	620 × 10^6^	0.21	23.2
01-008	High	1670 × 10^6^	0.31	13.5
02-009	High	1380 × 10^6^	0.18	19.0

^1^Participant identification number.

^2^Vector copy number per cell.

Several tests were performed on blood samples collected from trial participants before and after AGT103-T cell product infusion. Initially, we assessed standard hematology markers including the white blood cell count, total lymphocyte count, absolute CD4+ T cell count and fraction of CD4+ T cells among total lymphocytes (Figure 2). We noted the nadir of lymphocyte count at Day 3 after infusion. We do not know if this represents a lingering effect of the conditioning regimen or a response to cell product infusion.

**Figure 3:**
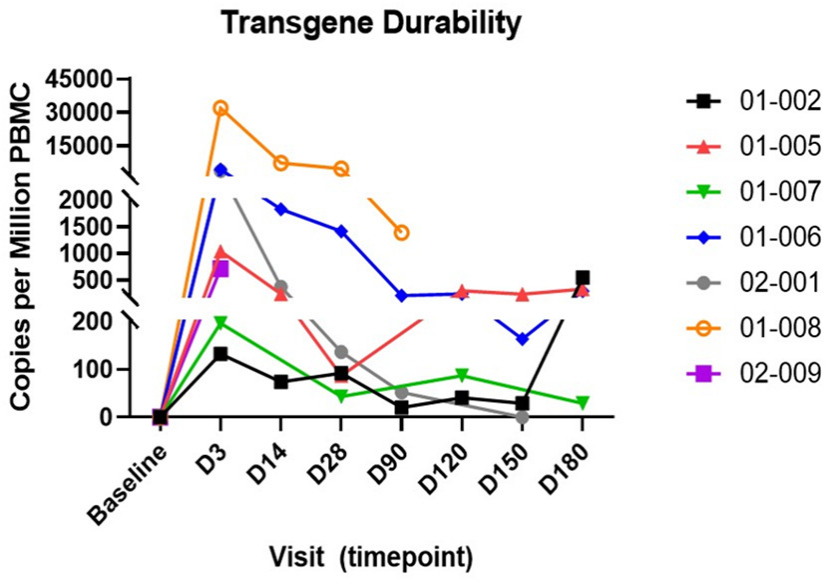
Persistence of genetically modified AGT103-T cells in participants post infusion. Transgene (AGT103 vector) durability is expressed as copies per million PBMC. Dashed line represents the lower limit of quantitation (LLOQ). Graph describes data up to the last visit for each participant. D3, D14…D180 represent number of days post infusion. Individual trial participants are indicated by a 5-digit identifier in the legend.

The durability of transduced cells was assessed by measuring the number of copies of the transgene in PBMC. The AGT103 (vector) transgene was detected in all participants up to their last monitoring visit, except for participant 02-001, when transgene was not detected in the sample collected at Day 150 after infusion (Figure 3). AGT103-modified cells peaked on Day 3 after infusion for all participants (range 132–31,882 per million PBMC; median 1,037 per million PBMC) and decreased thereafter. Samples collected during the last timepoint for monitoring (Day 180) had a median value of 439 transgene copies per million PBMC.

**Figure 4:**
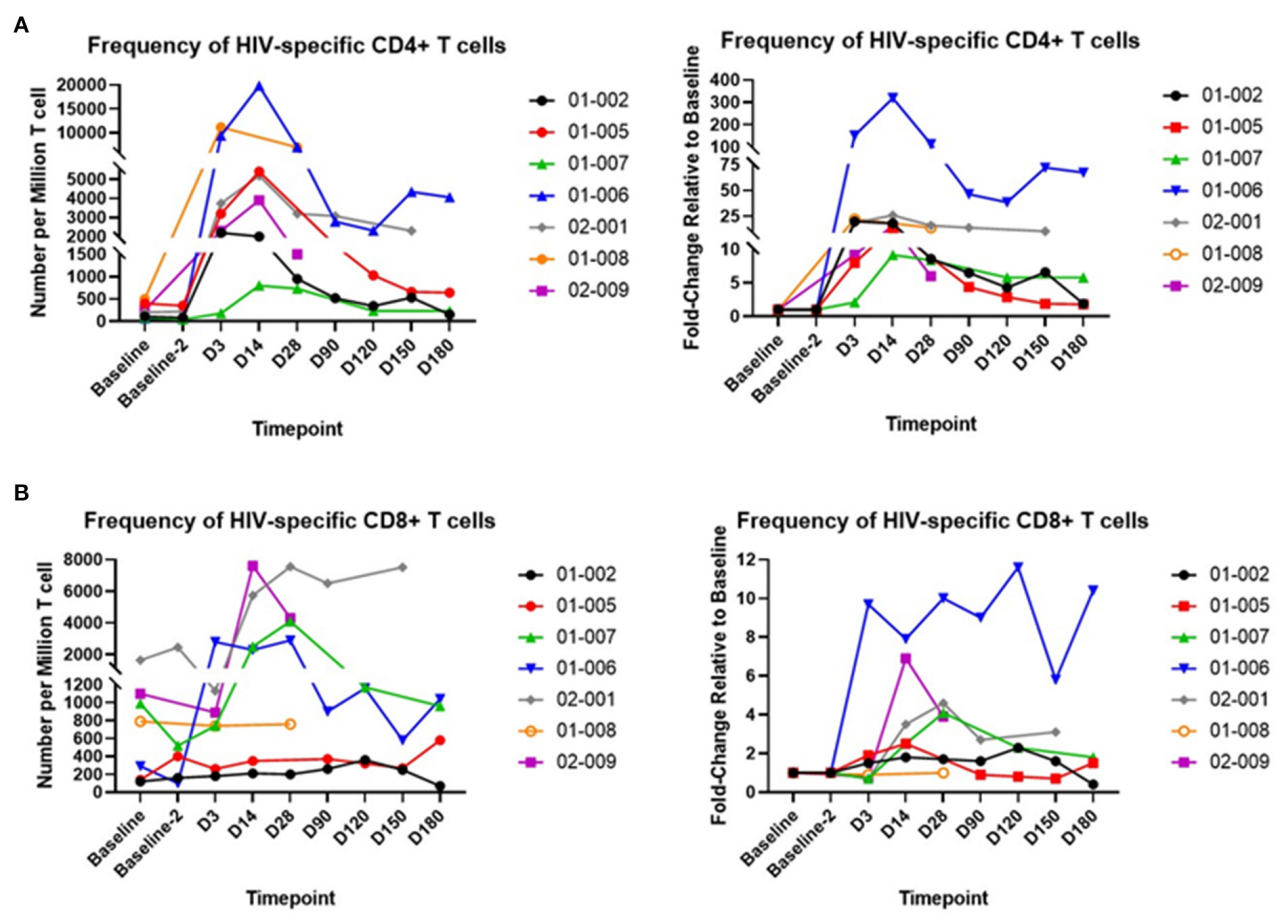
Response of purified peripheral blood CD4+ and CD8+ T cells to HIV Gag peptide stimulation *in vitro*. **(A)** Left panel, frequencies of CD4 T cells responding to Gag peptide, expressed as number per million CD3+ cells; right panel, fold change in numbers of Gag-specific CD4 T cells relative to baseline (for D3–D28) and baseline-2 (for D90–D180). **(B)** Left panel, frequencies of CD8 T cells responding to Gag peptide, expressed as number per million CD3+ cells; right panel, fold change in numbers of Gag-specific CD8 T cells relative to baseline (for D3–D28) and baseline-2 (for D90–D180). Individual trial participants are indicated by a 5-digit identifier in the legend.

We next evaluated CD4+ and CD8+ T cell responses to HIV Gag. Flow cytometry was used to measure cytokine responses in CD4+ or CD8+ T cells after Gag peptide stimulation. Data are reported as the frequency of cells with detectable intracellular Interferon-gamma after peptide stimulation in the presence of a Golgi blocker. The assay and the gating strategy is shown in Supplemental Figure 1. Collected blood samples were analyzed in two batches: one from baseline (before infusion) to Day 28 after infusion and a second batch representing Days 90–180 after infusion. Given the low frequency of HIV-specific T cells (< 0.02% of CD3+ T cells) in HIV+ participants who are ART-suppressed, a second baseline sample (baseline-2) was analyzed along with Day 90 through Day 180 samples to distinguish expected lower signals at later timepoints from baseline values. Gag-specific CD4+ T cells (Figure 4A) were detected at significantly higher levels on Day 3 after infusion compared to baseline. The median number of Gag-specific CD4+ T cells per million T cells on Day 3 after infusion was 3,200 (range 180–11,180), compared to baseline values with a median of 200 Gag-specific CD4+ T cells per million CD3+ T cells (range, 62–500). The proportion of Gag-specific CD4+ T cells peaked by Day 14 after infusion (median 4,545, range 800–19,800 Gag-specific CD4+ T cells per million T cells). The relative change in Gag-specific CD4+ T cells on Day 14 ranged from approximately 9–320, and gradually declined after Day 28. We observed approximately 2 to 70-fold increases (relative to baseline) in blood samples collected on Day 180 after infusion (Figure 4A, right panel). We also observed a gradual increase in Gag-specific CD8+ T cells that peaked on Day 28 after infusion (media 3,500, range 200–7,550 Gag-specific CD8+ T cells per million T cells) relative to baseline (median 790, range 120–1,650 Gag-specific CD8+ T cells per million T cells) (Figure 4B). However, the peak change relative to baseline (1.7 to 10-fold at Day 28 post infusion) was not as striking as the relative increase in Gag-specific CD4+ T cells (Figure 4A). This is not surprising given that CD8+ T cells are depleted during AGT103-T manufacturing, and Gag-specific CD8+ T cells among all peripheral blood CD8+ T cells comprise a relatively small fraction (< 0.5%, data not shown).

## Discussion

Our study showed that autologous AGT103-T T cell therapy products were safe and well tolerated in study participants without serious adverse events. We demonstrated persistence of AGT103-modified T cells and elevated Gag-specific CD4+ T cells that up to 180 days after infusion. Our results indicate that AGT103-T cell product infusion altered antiviral immunity in trial participants and transiently increased Gag-specific T cell responses.

A critical goal in this Phase 1 study was to demonstrate safety for an autologous cell product that was highly enriched for Gag-specific CD4+ T cells. In addition to standard panels for adverse and significant adverse event reporting, we identified three specific risks for persons receiving the AGT103-T cell product: (1) cytokine release syndrome (CRS), (2) immune effector cell-associated neurotoxicity (ICANS), and (3) immune reconstitution inflammatory syndrome (IRIS). CRS manifests as uncontrolled or excessive release of soluble immune mediators with life-threatening toxicity (24, 25). CRS is a common adverse event during cell therapy and we adopted standard diagnostic and treatment approaches for responding to CRS if it occurred after AGT103-T cell infusion. To date, no treatment was required for CRS during our study. ICANS has a complex presentation but may also be life threatening. The onset of ICANS after cell infusion requires specific diagnostic criteria and pre-planned medical responses (24, 25), and was considered a potential stopping criterion in our clinical trial. Fortunately, ICANS was not observed in the AGT103-T cell study. IRIS is a well-known phenomenon in HIV disease (26) that results from rapid rises in CD4+ T cell counts after successful antiretroviral therapy (27) and may manifest as strong responses to opportunistic infections when pathogen immunity is restored by antiretroviral therapy (28). IRIS was not observed among participants in our Phase 1 trial.

All other AEs reported were mild (Grade 1 and 2) and resolved within 1–2 days. The most frequent side effect was nausea after cyclophosphamide conditioning, a common side effect of cyclophosphamide even at low doses that were used in this study (29). Initially the rationale for cyclophosphamide conditioning was to enhance engraftment of genetically modified T cells (30). However, a recent study evaluated multiple doses of cyclophosphamide to enhance engraftment of an adoptive T cell therapy for HIV, and observed no significant difference in engraftment between cohorts that did or did not receive cyclophosphamide prior to infusion (31). We observed that WBC counts continued to decrease until Day 3 after infusion. Given that the half-life of cyclophosphamide is in hours (32), the most likely explanation for this continuous decrease in WBC counts after infusion is a prolonged cytostatic effect of cyclophosphamide. In a recent study, cyclophosphamide conditioning only 2 days prior to infusion (31) did not improve engraftment. Thus, re-evaluation of cyclophosphamide conditioning is warranted for future studies. The most common infusion-related AEs were headache and body aches, which were definitively related to infusion in only 1 out of 7 (14%) participants. The patterns of AEs observed here were similar to a previous study evaluating safety and efficacy of genetically modified T cells for HIV disease (31).

Another goal was to show that genetically modified AGT103-T cells would persist after infusion. The number of AGT103-modified cells was highest in the first sample collected post infusion (Day 3) and declined thereafter. The rank order of gene marked cells per dose was the same as the rank order of transgene copies per million cells, indicating a dose response relationship for this cell product. Similar patterns of cell engraftment were observed for other gene modified T cell therapies for HIV (21, 33), although those studies generally reported higher levels of marked cells in blood compared with our results. Higher detection of engraftment can be attributed to the use of multiple cell infusions, relatively larger number of cells per dose, or the methods used for calculating cell dose (21, 33, 34). Additionally, decreases in the fraction of AGT103-modified T cells are not surprising. Lymphocytes counts returned to pre-conditioning levels during the first weeks after infusion and declines in the proportions of genetically marked cells are observed routinely in gene therapy studies (21, 31, 33, 35).

Sustaining and enhancing CD4+ T cell function is critical for reconstituting immunity in HIV infection. A hallmark of AGT103-T cell products is the enrichment and expansion of HIV-specific CD4+ T cells (10) that has not been achieved in other products tested to date. We sought to measure CD4+ and CD8+ T cell responses to HIV Gag protein at various timepoints after infusion with AGT103-T. Unlike the number of AGT103-modified cells, that peaked by 3 days after infusion, peak levels of Gag-specific CD4+ T cells occurred around 14 days after infusion. This observation may be explained by proliferation of Gag-specific CD4+ T cells in 5 out of 7 (71%) infused participants. The highest relative increases in Gag-specific CD4+ T cells from Days 3 to 14 after infusion were observed in participant 01-007 (4-fold increase) and participant 01-006 (2-fold increase). We also observed an initial increase in Gag-specific CD8+ T cells per million T cells between Days 3 and 28 after infusion. However, the increase relative to baseline was less pronounced among CD8+ T cells compared to Gag-specific CD4+ T cells. Nevertheless, these increases in the number of HIV-specific CD4+ and CD8+ T cells up to Day 28 after infusion are intriguing, given participants are ART-suppressed and viremia remained undetectable throughout the observation period.

It is important to note that increased numbers of antigen specific CD4+ T cells exceeded the number of genetically marked cells. Consequently, infusion of the AGT103-T cell product stimulated a response among non-marked (normal) cells including CD8+ T cells. Whether this reflects an effect of the gene marked cells on host immunity or another mechanism is not resolved and remains under study.

We have noted the presence of extracellular Gag protein in the final cell product despite the absence of infectious HIV (data not shown). We believe this protein is mainly Gag p24 capsid protein that was present in the lentivirus stock used for cell transduction and was not removed even after extensive cell washing prior to cryopreservation. Whether Gag protein infused along with the cell product can initiate T cell responses is unknown and additional animal studies are needed to resolve this issue. It is important to note that the increases in Gag-specific T cell responses observed after AGT103-T infusion are substantially greater than changes observed after therapeutic immunization [e.g. (36)] and we do not expect high immunogenicity for antigens delivered by intravenous route. Consequently, we favor the explanation that Gag-specific T cell responses present after infusion reflect the positive impact of a substantial dose of Gag-specific CD4+ T cells. The observed changes after AGT103-T cell infusion document an objective clinical response to AGT103-T cell therapy even if they do not prove the mechanism of action.

The current study has several limitations. The number of enrolled and successfully infused participants is small, which impedes data analysis and interpretation. Detection of genetically modified cells and Gag-specific T cells was limited to peripheral blood samples because the protocol for this initial study did not include mucosal or lymph node biopsy specimens. Finally, we employed a conservative plan for clinical sampling after cell infusion and some studies, including detailed evaluation of T cell phenotype markers, were not possible due to limitations in the available material.

Multiple cell and gene therapy products were developed for HIV therapy (37) and several groups have reported recently on new approaches. The bacterial MazF endoribonuclease has higher activity against HIV RNA compared to host RNA and was expressed in CD4+ T cells that were infused as an autologous product (38). Treatment was well tolerated but all individuals experienced rebound viremia upon antiretroviral treatment interruption suggesting that simply protecting a fraction of bulk CD4+ T cells from HIV depletion, was insufficient to provide control over viremia. This result is similar to previous efforts that used antisense RNA (35, 39) or zinc-finger endonuclease deletion of CCR5 genes (31) to modify bulk CD4+ T cells. Efforts to improve antiviral immunity have also genetic modification of T cells. Chimeric antigen receptors expressed on T cells (CAR-T) were developed using broadly neutralizing antibodies, were capable of lysing HIV-expressing cells (40), and could be protected from HIV-mediated depletion by inserting the expression construct into the CCR5 gene locus (41). Using a combination of two neutralizing antibodies a duoCAR-T cell product achieved superior inhibition of HIV replication through simultaneous recognition of two sites on the envelope glycoprotein of HIV (42). The duoCAR-T approach is being tested in a current Phase 1/2 clinical trial (NCT04648046, www.clinicaltrials.gov).

While cell and gene therapies for HIV have been safe and well-tolerated, none so far have succeeded in achieving durable control over viral replication, with the results from duoCAR-T still pending. We believe an opportunity is missed when autologous cell products consist of bulk CD4+ T cells and are not enriched for the HIV-specific subset and especially the Gag-specific CD4+ T cells, which we know to be the critical for controlling HIV disease. The CD4+ T cell response to Gag is correlated with natural virus suppression including elite controllers, while a preponderance of CD4+ T cells specific for Envelope glycoprotein is linked to unfavorable outcomes (2), and levels of virus-specific CD8+ T cell responses are generally not predictive of clinical status (43, 44).

Strong Gag-specific CD4+ T cell responses are associated with control of viremia (3, 17) and natural virus suppression including elite virus control in adults or children (5, 7, 45–47). Elite controllers may also have lower levels of cell surface CCR5 receptor resulting in viral resistance (48) and they have lower genetic diversity among HIV sequences (49) suggesting that antiviral immunity, including effective CD8+ CTL function, has contained virus and prevented the evolution of immune escape variants. We do not disregard the critical importance of innate immunity and B cell immunity for virus control, but many of these responses are directly or indirectly affected by the action of antigen specific CD4+ T cells. Thus, many studies encourage a view that potent CD4+ T cell immunity against HIV may be key to natural virus control. The AGT103-T cell product was envisioned as an immunotherapy for HIV disease that depended on reconstituting a critical component of HIV immunity and preventing its destruction during any subsequent rise in viremia. We expect that reconstituted and durable Gag-specific CD4+ T cells will provide necessary help for B cells to improve the production of potent, neutralizing antibodies, and will support continuous differentiation of CD8+ T cell clones capable of recognizing and suppressing cells expressing viral escape variants. The encouraging initial results from this Phase 1 study suggest further clinical studies are warranted.

## Data availability statement

The datasets presented in this article are the property of American Gene Technologies International, Inc., and will be released through this and subsequent publications. Requests to access the datasets should be directed to JG, jgalvin@americangene.com.

## Ethics statement

The studies involving human participants were reviewed and approved by Institutional Review Board of Advarra, Inc., and the Institutional Review Board of Georgetown University Medical School. The patients/participants provided their written informed consent to participate in this study.

## Author contributions

NM qualified and performed assays and drafted and edited the manuscript. HL and TL developed assays and interpreted data and edited the manuscript. JG provided funding and reviewed the manuscript. PK and JB contributed to development of the clinical trial protocol, recruited and treated participants, interpreted data, and reviewed the manuscript. CP contributed to assay development, reviewed and interpreted data, and wrote and edited the manuscript. All authors contributed to the article and approved the submitted version.

## Funding

The authors declare that this study received funding from American Gene Technologies International, Inc. The funder was involved in study design, data collection, analysis and interpretation of the data, writing, and submitting the article for publication.

## Conflict of interest

Authors HL, TL, and CP are shareholders in American Gene Technologies International, Inc. Author JG is a shareholder and current employee of American Gene Technologies, International, Inc. Authors PK and JB received funding for clinical trial costs from American Gene Technologies International, Inc. Authors HL and CP are current employees of Viriom Inc. Author NM is a current employee of BioNTech.

## Publisher's note

All claims expressed in this article are solely those of the authors and do not necessarily represent those of their affiliated organizations, or those of the publisher, the editors and the reviewers. Any product that may be evaluated in this article, or claim that may be made by its manufacturer, is not guaranteed or endorsed by the publisher.

## Supplementary material

Supplementary Figure 1 **(A)** Assay workflow for evaluating T cell response to Gag peptides. **(B)** Flow cytometry gating strategy and representative results for CD8+ or CD4+ T cells expressing Interferon gamma.
