# Supplementary figures and images for: Safety and durability of AGT103-T autologous T cell therapy for HIV infection in a Phase 1 trial
Source: Front Med (Lausanne). 2022 Nov 14;9:1044713. doi: 10.3389/fmed.2022.1044713 (PMC9701732; doi:10.3389/fmed.2022.1044713)

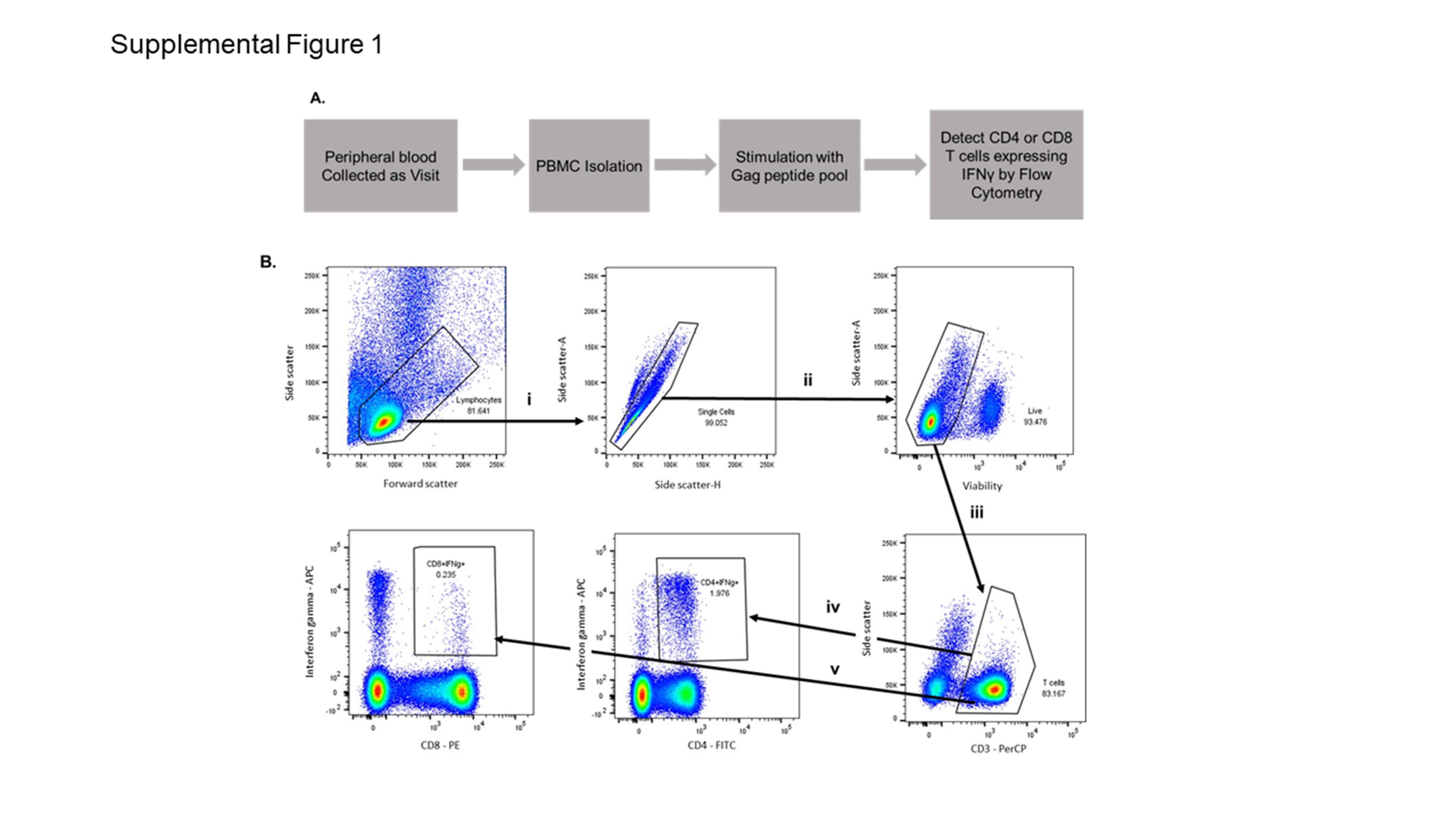

Supplement: Supplementary Figure 1 — (A) Assay workflow for evaluating T cell response to Gag peptides. (B) Flow cytometry gating strategy and representative results for CD8+ or CD4+ T cells expressing Interferon gamma. [file Image_1.TIF]
